# Calsyntenin-1 Promotes Doxorubicin-induced Dilated Cardiomyopathy in Rats

**DOI:** 10.1007/s10557-022-07389-x

**Published:** 2022-11-09

**Authors:** Mingxiang Zhu, Yibing Chen, Liting Cheng, Xin Li, Yanying Shen, Ge Guo, Xiang Xu, Hanlu Li, Hao Yang, Chunlei Liu, Kunlun He

**Affiliations:** 1 Medical School of Chinese PLA, Beijing, 100853 China; 2 Medical Big Data Research Center, Chinese PLA General Hospital, Beijing, 100853 China; 3 Translational Medicine Research Center, Medical Innovation Research Division of Chinese PLA General Hospital, Beijing, 100853 China; 4 School of Medicine, Nankai University, Tianjin, 300071 China; 5 Department of Radiation Oncology, Inner Mongolia Cancer Hospital and Affiliated People’s Hospital of Inner Mongolia Medical University, Huhhot, China

**Keywords:** Calsyntenin-1, Cardiotoxicity, Doxorubicin, Dilated cardiomyopathy

## Abstract

**Purpose:**

Doxorubicin is an important cancer chemotherapeutic agent with severe cardiotoxic effects that eventually lead to dilated cardiomyopathy (DCM). Calsyntenin-1(CLSTN1) plays a critical role in the nervous system, but its relevance in cardiovascular diseases is unknown. We investigated the significance of CLSTN1 in doxorubicin-induced DCM.

**Methods:**

CLSTN1 expression in doxorubicin-induced DCM rats and H9c2 cells was determined using western blotting. To further explore the functions of CLSTN1, a cardiac-specific CLSTN1 overexpression rat model was constructed. The rats were subjected to analysis using echocardiographic, hemodynamic, and electrocardiographic parameters. Potential downstream molecules in CLSTN1 overexpression heart tissue were investigated using proteomics and western blotting. Finally, a knockdown of CLSTN1 was constructed to investigate the rescue function on doxorubicin-induced cell toxicity.

**Results:**

CLSTN1 protein expression increased drastically in doxorubicin-induced DCM rats and H9c2 cells. Under doxorubicin treatment, CLSTN1 protein-specific overexpression in the heart muscle promoted cardiac chamber enlargement and heart failure, while the knockdown of CLSTN1 reduced doxorubicin-induced cardiomyocyte toxicity in vitro. At the mechanistic level, overexpression of CLSTN1 downregulated SERCA2 expression and increased the phosphorylation levels of PI3K-Akt and CaMK2.

**Conclusion:**

Our findings demonstrated that CLSTN1 promotes the pathogenesis of doxorubicin-induced DCM. CLSTN1 could be a therapeutic target to prevent the development of doxorubicin-induced DCM.

**Supplementary Information:**

The online version contains supplementary material available at 10.1007/s10557-022-07389-x.

## Introduction

Doxorubicin (Dox) is an anthracycline-class chemotherapeutic agent that is frequently used in oncology [1]. However, mounting evidence suggests that Dox directly triggers cardiotoxicity, thereby limiting its clinical utility. Accumulation of Dox in the heart tissue may cause myocardial injury, apoptosis, and oxidative damage, ultimately resulting in dilated cardiomyopathy (DCM) [2, 3]. For decades, numerous studies have been conducted with a focus on the molecular mechanism of Dox-induced DCM to prevent or reduce this cardiotoxicity.

Calsyntenin-1 (CLSTN1), a type I transmembrane protein, is a member of the cadherin superfamily of cell-adhesion molecules. Although the brain displays the highest levels of expression, CLSTN1 can also be detected in many other tissues, such as the heart, skeletal muscle, kidneys, and placenta [4, 5]. This protein can bind to Ca^2+^ with its cytoplasmic domain and may even modulate Ca^2+^-mediated postsynaptic signals [5]. Previous research has revealed that CLSTN1 regulates the PI3K-Akt pathway by controlling the axonal localization of the insulin receptor DAF-2c [6]. Additionally, the cytoplasmic domains of CLSTN1 directly interact with kinesin-1 to regulate anterograde transport [7]. CLSTN1 can also enhance the X11-like protein-mediated stabilization of amyloid beta-protein precursor metabolism and reduce the generation of neurotoxic β-amyloid peptides in brain tissue [8–10]. Recently, CLSTN1 has been identified as a potential pathogenic gene in ischemic cardiomyopathy [11, 12]. However, its role and related mechanisms in Dox-induced DCM remain unclear.

According to previous studies, Dox seriously disrupts calcium homeostasis in the heart, which then decreases the systolic contraction of the cardiac myocytes [13, 14]. The resultant cardiac injury is significantly regulated by the PI3K-Akt pathway [15]. Therefore, by regulating the calcium and PI3K-Akt pathways, we aim to understand the association of CLSTN1 with Dox.

In this study, we demonstrated the role of CLSTN1 as a key determinant in cardiotoxicity. CLSTN1 was shown to contribute to the pathogenesis of DCM via the regulation of phosphorylation of CaMK2 and PI3K-Akt in rat models. CLSTN1 could, therefore, be a therapeutic target to prevent the development of Dox-induced DCM and heart failure.

## Material and Methods

### DCM Rat Model

Wild-type (WT) Sprague Dawley (SD) rats were obtained from the Experimental Animal Centre of the General Hospital of the PLA (Beijing, China). All experiments were conducted according to the guidelines issued by and with the approval of the Institutional Animal Care and Use Committees of the Chinese PLA General Hospital. All animals were kept at the Animal Centre of the General Hospital under specific pathogen-free conditions.

Based on a former study, tail vein injections of Dox were performed to establish the DCM model [16–19]. The SD rats (male, 8 weeks old; 250–300 g; *n* = 18) were randomly assigned to a control (Con) group (*n* = 9) and a DCM group (*n* = 9). Each rat in the DCM group was administered a cumulative dose of 18 mg/kg Dox (doxorubicin hydrochloride; Solarbio, Beijing, China) in six consecutive intravenous cycles for one month (injected once every five days) by injection into the tail vein. The control group received injections of the same volume of saline at the same time points. Transthoracic echocardiography was performed one week after the injection to evaluate heart function while under deep anaesthesia with pentobarbital sodium (50 mg/kg, Sigma, USA). The rats were then exsanguinated by cardiac puncture, and the hearts were harvested for subsequent experiments.

### CLSTN1 Overexpression Rat Model

CLSTN1 overexpression (CLSTN1-OE) transgenic Sprague Dawley (SD) rats were purchased from Biocytogen Pharmaceuticals Co., Ltd. (Beijing, China). The information on CLSTN1 was obtained from the NCBI database (Ensembl gene ID 313717 and RefSeq transcript ID NM_001007092.1). The floxed CLSTN1 allele was generated by injecting Cas9/sgRNA-embedded CLSTN1 vector into SD rat blastocysts. Chimeras were then bred into a wild-type SD rat strain to generate rats with stable CLSTN1 expression.

Heart-specific CreERT transgenic rats were obtained from Biocytogen Pharmaceuticals Co., Ltd. (Beijing, China) and the transgene construct of the Cre gene has been previously described [20]. The CLSTN1 floxed rats were crossed with the heart-specific Cre transgenic rats. Conditional activation of Cre recombinase was induced by intraperitoneal administration of tamoxifen (MedChemExpress, Princeton, NJ, USA), resulting in cardiac-specific CLSTN1 overexpression. Genotypes were determined by polymerase chain reaction (PCR) using tail snip DNA, four weeks after birth. All experimental rats (male, 6 weeks old; 150–250 g) received intraperitoneal tamoxifen (55 mg/kg per day) for five consecutive days.

There were two groups in the first experiment: WT and CLSTN1 OE (*n* = 6 per group). Transthoracic electrocardiograms were recorded before the first time and then monthly after tamoxifen administration for two months. In the second experiment, the rats were divided into four groups a week after tamoxifen injection: Control-Wild type group (Con-WT; *n* = 13), Control-CLSTN1 overexpression group (Con-CLSTN1 OE; *n* = 10), DCM-Wild type group (DCM-WT; *n* = 13), and DCM-CLSTN1 overexpression group (DCM-CLSTN1 OE; *n*=13), based on their genotypes and subsequent Dox injection. The rats were injected with Dox or an equivalent volume of saline via tail veins, as described in Section 2.1. After the DCM model was established, the rats underwent transthoracic echocardiographic, hemodynamic, and electrocardiogram (ECG) analyses while under anaesthesia. Then, the rats were killed by cardiac exsanguination, and the organs were immediately weighed and harvested for subsequent experiments.

### Transthoracic Echocardiographic Analysis

Heart function was assessed 1 week after Dox injection with a Vevo2100 System (30 MHz scan head; VisualSonics Inc., Toronto, ON, Canada). The rats underwent transthoracic echocardiography under anesthesia. M-mode echocardiography of the left ventricle was performed along the long axis to assess left ventricle function, as previously described [21]. Data were analyzed using VisualSonics software suite. The diastolic left ventricular anterior wall thickness (LVAWTd), systolic left ventricular anterior wall thickness (LVAWTs), diastolic left ventricular posterior wall thickness (LVPWTd), systolic left ventricular posterior wall thickness (LVPWTs), left ventricular end-diastolic diameter (LVEDD), left ventricular end-systolic dimension (LVESD), left ventricular end-diastolic volume (LVEDV), left ventricular end systolic volume (LVESV), LV ejection fraction (EF), and fractional shortening (FS) were averaged and determined from three cardiac cycles.

### Hemodynamic and ECG Analysis

After completing the echocardiographic analysis, the anesthetized rats underwent hemodynamic and ECG analysis in the closed chest using PowerLab data acquisition units A PowerLab Chart data acquisition system (ADInstruments, Colorado Springs, CO, USA.) for small animals equipped with an SPR-838 Millar Mikro tip catheter. The right carotid artery was exposed and the pressure transducer was inserted retrogradely into the artery and finally into the left ventricle. Data were acquired using LabChart8 software.

ECGs were recorded by inserting needle electrodes into the rat limbs at the same time during hemodynamic analysis. Surface ECG intervals were measured using a LabChart8 system (AD Instruments, New Zealand)). ECG analysis was performed using the Hamilton–Tompkins QRS detection algorithm.

### Histological Analysis

The rat hearts were fixed in 10% phosphate-buffered formalin and embedded in paraffin. Hearts were sectioned into slices using a Leica RM2016 microtome (Leica MZ10 F, Leica, Wetzlar, Germany).

Hematoxylin and eosin (HE) staining (G1003, HE dye solution set, Servicebio Technology, Wuhan, China) and Masson staining (G1006, Masson dye solution set, Servicebio Technology, Wuhan, China) were performed on paraffin-embedded heart sections. Immunofluorescence was performed using antibodies against CLSTN1 (Calsyntenin-1 Polyclonal antibody, ProteinTech, Wuhan, China) and horseradish peroxidase-conjugated anti-rabbit secondary antibodies (G1215, Immunohistochemistry Kit, Servicebio Technology, Wuhan, China). Next, 3,3’-diaminobenzidine was added to develop the color, and the positive was brownish yellow. Images were captured using an upright optical microscope (Nikon, Eclipse E100e, Japan).

For terminal deoxynucleotidyl transferase dUTP nick end labeling (TUNEL) analysis, heart sections were incubated at room temperature for 2h using a TUNEL assay kit (G1501, Servicebio Technology, Wuhan, China). The cells were then incubated with a 4',6-diamidino-2-phenylindole (DAPI) (G1012, Servicebio Technology, Wuhan, China) solution at room temperature for 10 min. Immunofluorescence staining was then performed. The antibody against CLSTN1 was incubated with the slides overnight at 4°C. Cy3 conjugated secondary antibody (GB21303, Cy3 conjugated Goat anti-rabbit IgG; Servicebio Technology, Wuhan, China) was incubated at room temperature for 50 min in the dark. The DAPI solution was then incubated at room temperature for 10 min in the dark, and the spontaneous fluorescence quenching reagent was added and incubated for 5 min. Microscopic examination and image collection were performed using a fluorescence microscope (Nikon, Nikon Eclipse C1, Japan).

### Cell Culture

H9c2 cells were grown in Dulbecco’s modified Eagle’s medium (DMEM, GIBCO, USA) supplemented with 10% Foetal Bovine serum (FBS, GIBCO, USA) at 37°C and 5% carbon dioxide (CO_2_), and passaged at 70–80% confluence. Before the treatment, cells were starved for 12 hours in DMEM without FBS and were then incubated with Dox (0 μM, 0.1 μM, 0.3 μM, 0.6 μM, 1 μM) for 24 h to induce cardiomyocyte injury and thereby construct a Dox-induced DCM model. Cell activity was measured using cell morphology and CCK8 (Dojindo laboratories, Shanghai, China), while protein expression levels were determined using western blotting.

### Knockdown of CLSTN1 in H9c2 Cells

Lentiviral vectors were constructed by Genechem Co (Shanghai, China), and were employed to implement CLSTN1 knockdown in H9c2 cells. Both the CLSTN1-knockdown lentivirus and a negative control lentivirus (NC-lentivirus, Genechem Co.) were prepared and tittered to 10^8^ transfection units per milliliter. Cell activity was measured using CCK8 (Dojindo laboratories, Shanghai, China). Protein expression levels were detected using western blotting.

### Western Blotting

Heart tissues were homogenized in liquid nitrogen and the homogenate was lysed on ice for 30 min using enhanced RIPA lysis buffer (Enhanced RIPA Lysis Buffer C1053+, Applygen Technologies, Beijing, China). Cell proteins were directly collected using enhanced RIPA lysis buffer and lysed on ice for 1 h. Lysates (15–30 μg) of total protein were loaded per well and separated using a 10% SDS-PAGE gel. Primary antibodies were anti-CLSTN1 (Calsyntenin-1 Polyclonal antibody, 1:1000 dilution, ProteinTech, Wuhan, China); anti-GAPDH (GB12002, 1:2000 dilution, Servicebio, Wuhan, China); anti-phospho-CaMK2 (Anti-CaMK2 (phospho T286) antibody, ab171095, 1:2000 dilution, Abcam, Shanghai, China); anti- CaMK2 (Anti-CaMK2 antibody, ab52476, 1:2000 dilution, Abcam, Shanghai, China); anti-SERCA2 (Anti-SERCA2 ATPase, ab150435, 1:10000 dilution, Abcam, Shanghai, China); anti- phospho-PI3K p85 (Anti-PI 3 Kinase p85 alpha (phospho Y607), 1:1000 dilution, ab182651, Abcam, Shanghai, China); anti- PI3K p85(Anti-PI 3 Kinase p85 alpha, 1:1000 dilution, ab191606, Abcam, Shanghai, China); anti- phosphor-Akt (Phospho-Akt (Ser473) Rabbit mAb, 1:1000 dilution, 4060T, CST, Danvers, MA, USA); anti-Akt(Akt (pan) Rabbit mAb, 1:1000 dilution, 4691T, CST, Danvers, MA, USA); anti-VEGF(Anti-VEGF Receptor 2 antibody, 1:1000 dilution, ab39256, Abcam, Shanghai, China); anti- PI3 Kinase p110 beta(Anti-PI3 Kinase p110 beta, 1:1000 dilution, ab151549, Abcam, Shanghai, China); anti-caspase3(Anti-caspase3, 1:1000 dilution, ab32351, Abcam, Shanghai, China); anti-caspase9 (Anti-caspase9, 1:1000 dilution, ab32539, Abcam, Shanghai, China). The secondary antibodies were horseradish peroxidase (HRP)-goat anti-rabbit (G1213-100UL, Servicebio Technology, Wuhan, China) and HRP-goat anti-mouse (G1214-100UL, Servicebio Technology, Wuhan, China) secondary antibody. Pre-stained protein ladders (Spectra Multicolor High Range Protein Ladder, Thermo Fisher Scientific26625, UK) (3-Color Pre-stained Protein Standards, Accurate Biology, AG11919, China) provided the molecular weight mark

### Proteomics Data Analysis

A QExactive mass spectrometer (Thermo Fisher Scientific) was connected to an Ultimate3000 HPLC system for proteomic analysis. Whole heart tissues from Con-WT (*n* = 4), Con-CLSTN1 OE (*n* = 3), DCM-WT (*n* = 3), and DCM-CLSTN1 OE (*n* = 4) were isolated and homogenized in 2×Doc lysis buffer. After dissolution by sonication, the protein concentration of the supernatant was measured by BCA at a final concentration of 10 mM. Protein samples were then digested using the ProteoExtract digestion kit (Calbiochem, Gibbstown, NJ, USA) to obtain a 30kDa peptide.

The peptide samples were absorbed in 0.1% formic acid-water solution, and 100 ng of peptide was loaded onto a C18 reversed-phase precolumn (Thermo Fisher Scientific, USA) and separated with a 98-min linear gradient from 6% to 30% acetonitrile and 0.1% formic acid, at a flow rate of 500 nl/min. Full mass spectrometry (MS) scans were performed using an Orbitrap mass analyzer over a range of 350 to 1550 m/z with a mass resolution of 70,000. The automatic gain control (ACG) target was set at a value of 3e6 ions, and the maximum injection time was set at 50 ms.

In secondary MS, the top 20 parent ions were fragmented using high-energy collisional dissociation (HCD) with a collision energy of 27%. Fragmented parent ions were detected using an Orbitrap mass analyzer. The parameters used in the secondary MS were as follows: resolving power, 17,500; fixed first mass, 100; ACG target, 5e4 ions; maximum injection time, 50 ms; dynamic exclusion time, 20 s.

Data analysis was performed using Proteome Discoverer 2.1 (Thermo Fisher Scientific, USA) with the search engine Sequest. MS spectra were used to interrogate the Vitis proteins in the UniProt database (UniProt) (http://www.uniprot.org/). The following default settings were used for peptide/protein matching: the mass deviation of the parent ion was 20 ppm; the mass deviation of the secondary fragment ion was 0.05 Da; trypsin cleavage allowed up to two missing cleavages; protein acetyl (protein N-term), oxidation (M), and carbamidomethyl (C) were set as dynamic modifications; and false discovery rate (FDR) was calculated using the decoy database and was set to 1%. PCA was performed to obtain the principal coordinates from multidimensional data. The differentially expressed proteins were identified using ANOVA analysis with a threshold of fold change >2 or <0.5 and a *P* value < 0.05. Protein expression heatmaps were generated using Spotfire 7.7 (Perkin-Elmer). Differentially expressed proteins were mapped to the KEGG database (https://www.kegg.jp/kegg/pathway.html).

### Statistics Analysis

Data normality was tested using the Kolmogorov–Smirnov test. Results are expressed as mean ± SD (normal data) or median (interquartile range) (non-normal data). The Student’s T-test or Mann–Whitney nonparametric test was used for analysis between the two groups based on data normality. For multiple comparisons, ANOVA was followed by least significant difference (LSD) post-hoc test when the data were homogeneous, and the Mann–Whitney test, with a Bonferroni multiple comparison correction, was performed for non-homogeneous variances. SPSS (IBM SPSS Statistics, version 26) was used for the data calculation. A *P* value < 0.05 was considered statistically significant.

## Results

### CLSTN1 Upregulated in DCM Rats and H9c2 Cell Line

**Fig. 1:**
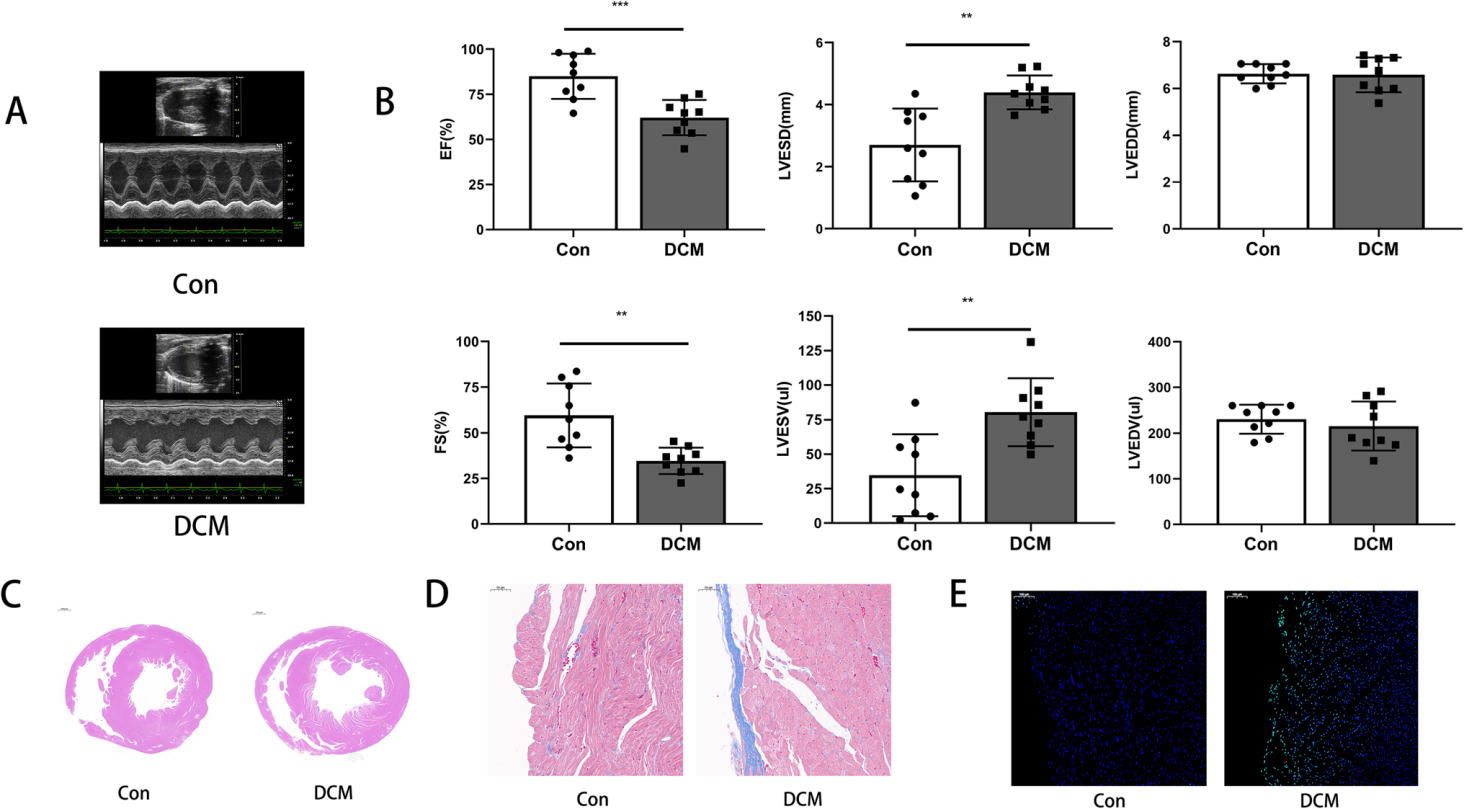
Dox-induced dilated cardiomyopathy model construction. Dilated cardiomyopathy (DCM) induced by Dox-injection in rats. **(A)** Representative M-mode echocardiographic images along the long axis of the heart. **(B)**Main echocardiographic parameters of heart structure and function. **(C–E)** Representative images of HE staining, Masson staining, and TUNEL assay. The green fluorescence indicates the TUNEL-positive cells, while the blue fluorescence indicates DAPI-staining nuclei. **P < 0.01, ***P < 0.001

We aimed to explore the effect of CLSTN1 expression on cardiotoxicity using a Dox-induced DCM rat model. First, the success of DCM models induced by Dox was verified. Representative echocardiographs showed contractile dysfunction and ventricular enlargement in the DCM model group (Fig. 1A). Echocardiographic results revealed that Dox injection significantly decreased ejection fraction (EF) and fractional shortening (FS), notably increasing the left ventricular end-systolic volume (LVESV) and left ventricular end-systolic diameter (LVESD), while it did not significantly alter the left ventricular end-diastolic volume (LVEDV) and left ventricular end-diastolic diameter (LVEDD) (Fig. 1B). Meanwhile, HE staining revealed enlarged ventricular diameter in the Dox-treated heart (Fig. 1C), Masson staining revealed increased fibrosis in the Dox-treated heart (Fig. 1D) and a TUNEL assay was performed to see increased apoptosis in the endocardial region in the Dox-treated heart (Fig. 1E). These results showed that DCM was induced in these rats by injection of Dox.

**Fig. 2:**
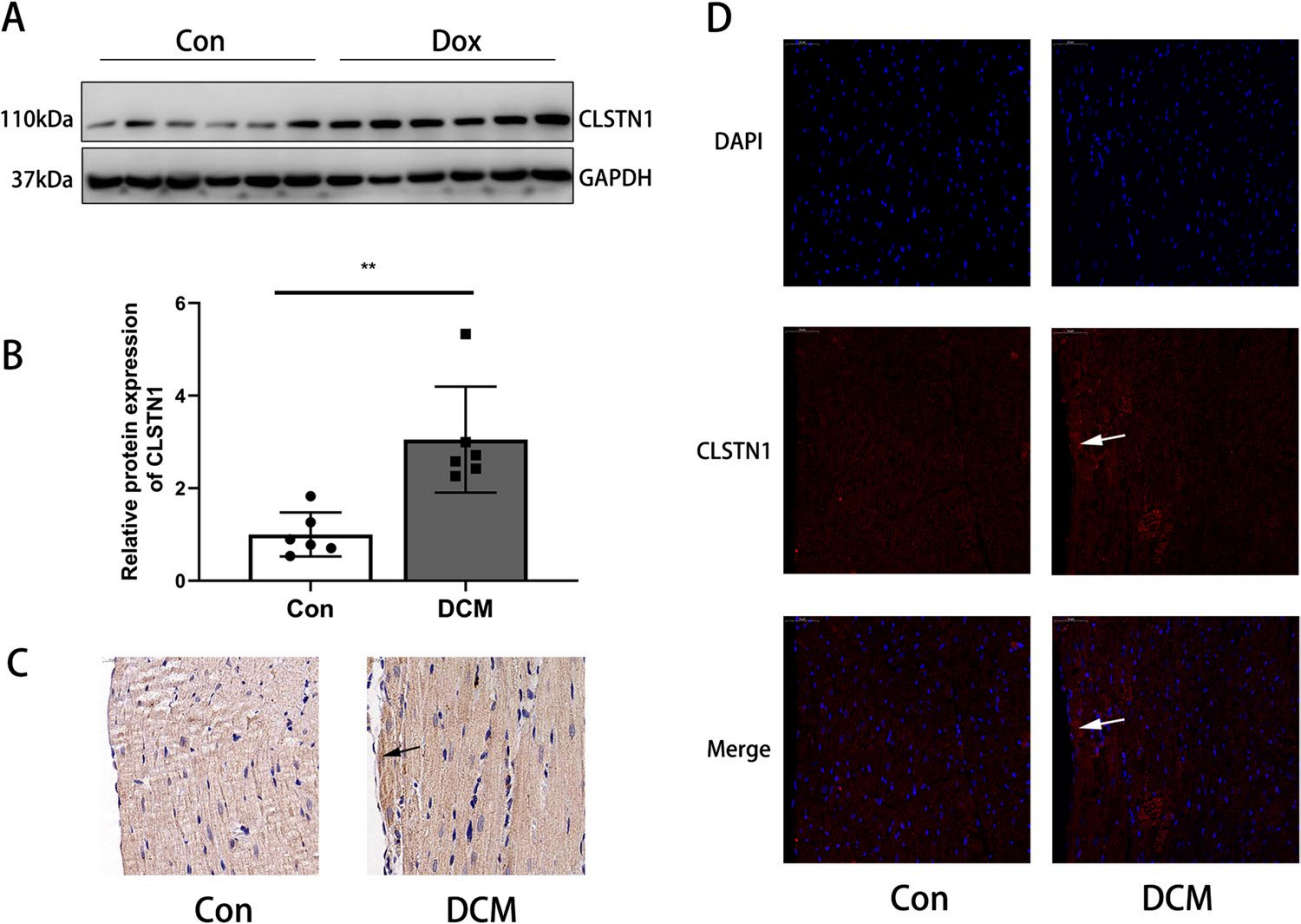
CLSTN1 Expression Level. High protein expression of CLSTN1 in the heart tissue of Dox-treated rats. **(A)** Images of western blot of CLSTN1 expression in rat hearts. (B) Protein quantification of CLSTN1 analysis by western blot relative to GAPDH. **(C)** Immunofluorescent labeling of DAPI (blue) and CLSTN1 (red) within cardiomyocytes. **(D)** Immunohistochemical staining of CLSTN1. **P < 0.01. The arrow shows the accumulation of CLSTN1 in myocardial cells

And then the expression of CLSTN1 in the control and DCM groups was measured. Western blot analysis revealed that the protein levels of CLSTN1 were significantly elevated in the Dox group more than in the control group with an approximately twofold increase in protein expression (Fig. 2A and  B). Immunofluorescence and immunohistochemical staining performed on heart sections demonstrated that CLSTN1 was widely expressed in the membranes of cardiomyocytes and the staining deepened after Dox injection to induce DCM (Fig. 2C and D).

**Fig. 3:**
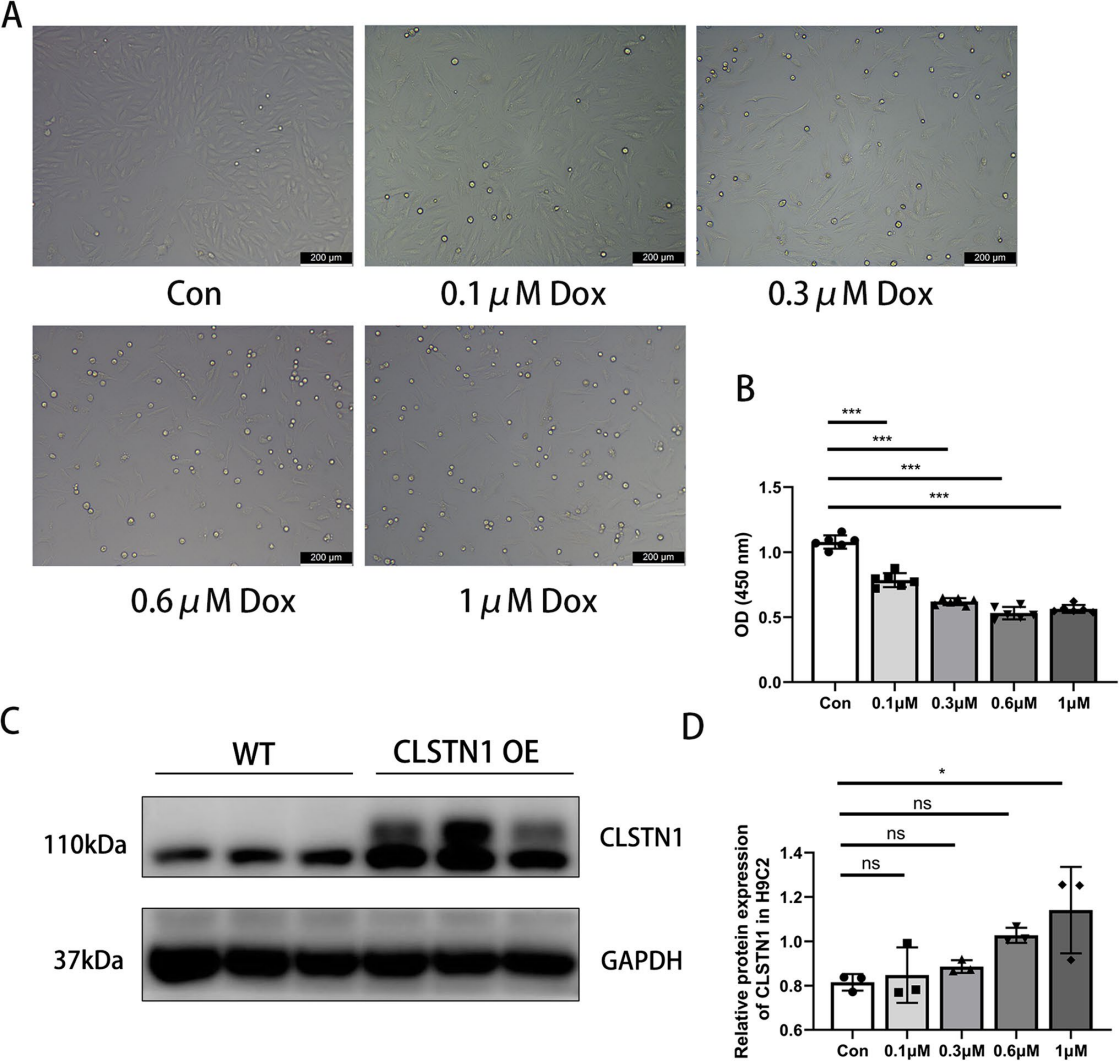
Dilated cardiomyopathy cell model. Dox-induced dilated cardiomyopathy model in H9c2 cells. **(A)** Images of morphology of H9c2 cells by light microscopy. **(B)** CCK8 assay results for cellular viability. **(C)** Images of western blot of CLSTN1 expression in H9c2. **(D)** Protein quantification of CLSTN1 by western blot relative to GAPDH

**Fig. 4:**
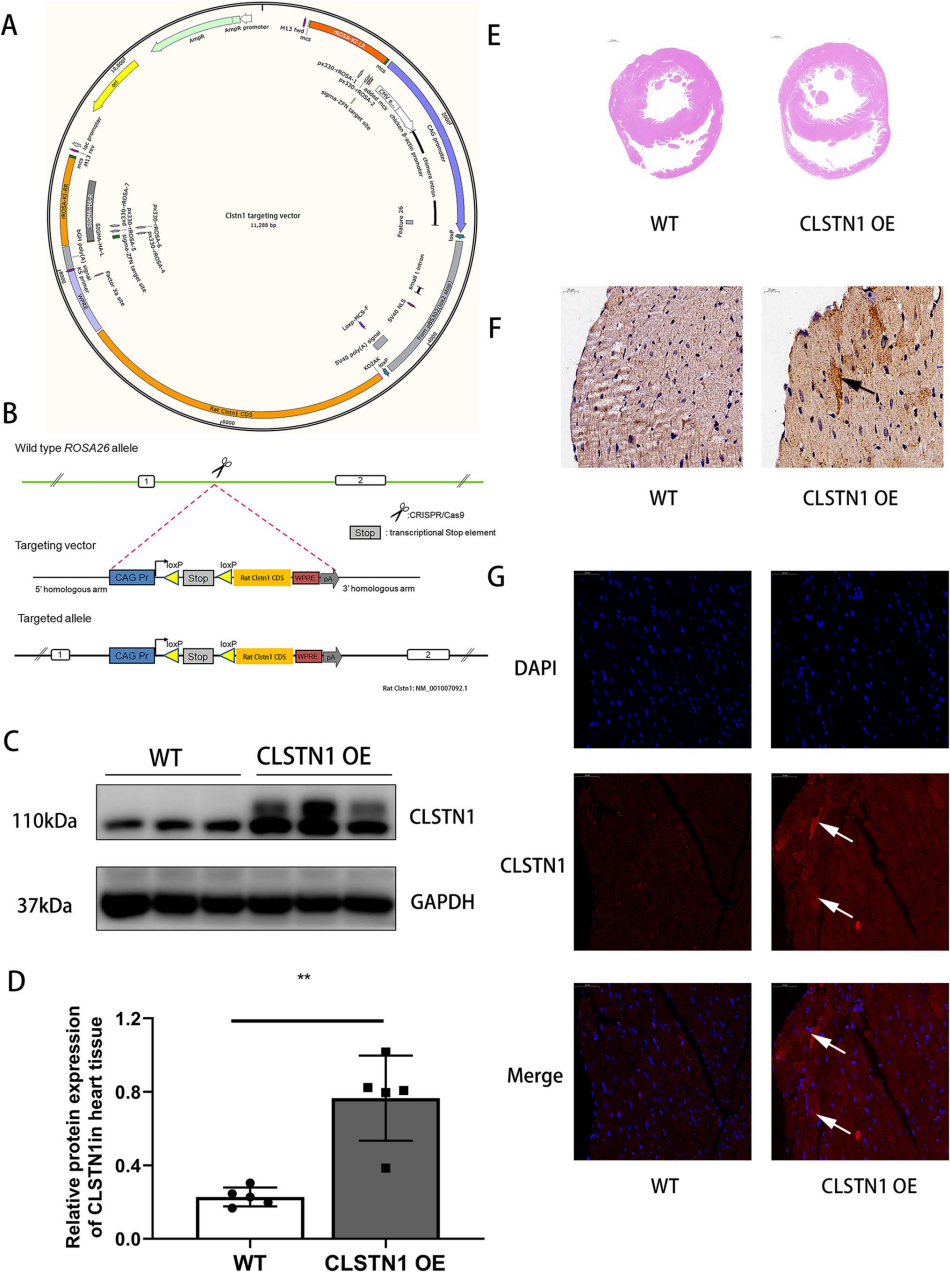
CLSTN1 overexpression in the rat heart tissue.** (A)** Schematic of CLSTN1 overexpression targeting vector. **(B)** The wild-type and CLSTN1 overexpression loci and gene targeting constructs. CAG promoter-loxP-Stop-loxP-rat CLSTN1 CDS-WPRE-pA targeting construct was inserted into Rosa26 targeted locus. **(C)** Western blot of CLSTN1 overexpression. **(D)** Statistical results for relative protein expression of CLSTN1 relative to GAPDH. **(E)** Representative HE staining results of the wild type and CLSTN1 overexpression heart tissues (scale bar = 20 μm). **(F-G)** Immunohistochemical and immunofluorescence images of CLSTN1 in heart tissues. Brown staining and particles indicate CLSTN1 protein expression in immunohistochemical staining (scale bar = 20 μm), while blue labeling indicates DAPI and red, CLSTN1 in cardiac immunofluorescent images (scale bar = 50 μm). CDS, coding sequence; CAG Pr, CAG promoter; loxP, loxP elements; Stop, stop codon sites. The arrow shows the accumulation of CLSTN1 in myocardial cells. **P < 0.01

We further validated the changes in CLSTN1 expression in vitro. H9c2 cells were incubated with different doses of Dox (0.1, 0.3,0.6, and 1 μM). As shown in Fig. 3A, Dox caused an increased loss of cardiomyocytes along with morphological changes in the cell. The results of the CCK8 assay demonstrated that the viability of cells decreased with an increase in the concentration of Dox in the culture medium (Fig. 3B) and that stimulating the cells with Dox for 24 h could induce toxicity in cardiomyocytes and thereby be used for subsequent experiments. The protein levels of calsyntenin-1 also significantly increased with Dox treatment, as is consistent with in vivo results (Fig. 4C and D).

### Use Tamoxifen-Inducible Cre/loxP System to Generate CLSTN1 Overexpression Model

We generated CLSTN1 conditional overexpression in the heart tissue using the Cre/loxP system. Using the CRISPR/Cas9 technique, the CLSTN1 knock-in (targeting) vector was constructed and used for microinjection into fertilized rat eggs **(**Fig. 4A). The knock-in mutation segmentation CAG Promoter-loxP-Stop-loxP-rat CLSTN1 CDS-WPRE-pA was inserted into the Rosa26 locus (Fig. 4B). CLSTN1 knock-in rats were crossed with Cre transgenic rats to generate CLSTN1 conditional overexpression in the heart tissue.

Total protein was isolated from various heart tissues for western blot analysis to confirm the upregulation of CLSTN1 expression in the heart (Fig. 4C and D). Cardiac histology was then performed. HE staining revealed ventricular enlargement in the CLSTN1 overexpression model compared to the wild type (Fig. 4E). Furthermore, based on immunofluorescence and immunohistochemical staining, this staining deepened, and the staining area increased in the overexpression groups (Fig. 4F and G). These results confirmed establishment of the CLSTN1 overexpression rat model.

### CLSTN1 Overexpression Aggravates Ventricle Dilatation and Heart Function

**Fig. 5:**
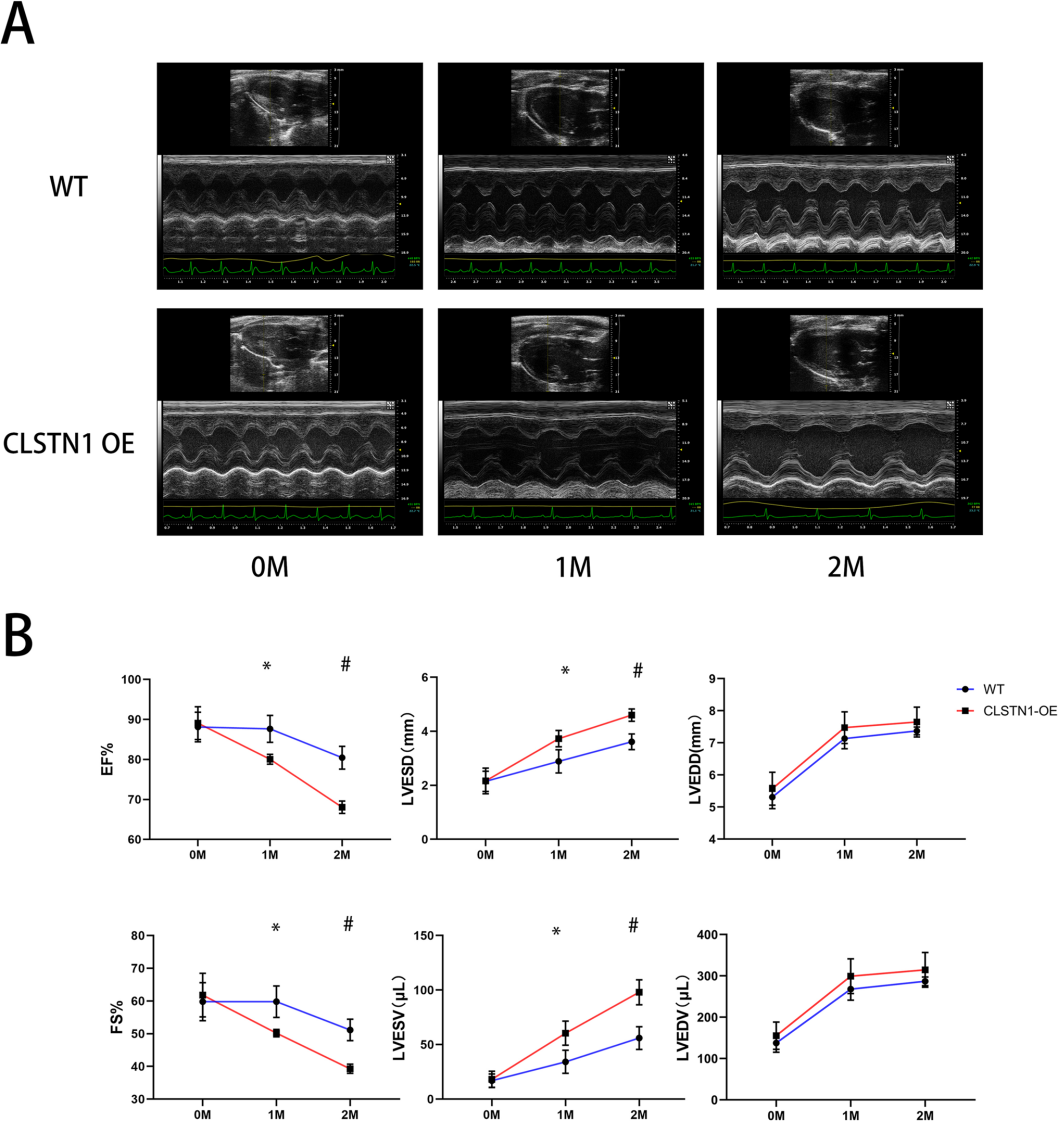
Serial echocardiographic measurements in WT and CLSTN1 overexpression rat models. Heart systolic function significantly worsened after CLSTN1 overexpression in the cardiomyocyte in a time-dependent manner. **(A)** Representative M-mode echocardiographic images along the long axis of the heart over time in WT and CLSTN1 OE group. **(B)** Changes in ultrasound indexes EF, FS, LVESD, and LVESV over time. Data are presented as mean ± SEM (*n* = 6 per group). 0 M indicates the start of the experiment; 1 M, 1 month after tamoxifen injection; 2 M, 2 months after tamoxifen injection. *P < 0.05 for WT group compared with CLSTN1 OE group in 1 month; #*P* < 0.05 for WT group compared with CLSTN1 OE group in 2 months

Subsequent experiments aimed to evaluate whether overexpression of CLSTN1 could influence the systolic and diastolic function of the heart. Echocardiography analyses were recorded before the first tamoxifen injections and then recorded every month afterward for 2 months. The results of the echocardiographic analysis showed that EF and FS alterations had markedly reduced, while LVESD and LVESV had significantly increased in CLSTN1 overexpression rats in a time-dependent manner. LVEDD or LVEDV were not significantly changed between the control group and overexpression group (Fig. 5A and B).

To further explore the role of CLSTN1 in Dox-induced dilated cardiomyopathy, Dox or an equal volume of saline was administered to the experimental rats. There were four groups in this experiment: Con-WT (*n* = 13), DCM-WT (*n* = 13), Con-CLSTN1 OE (*n*=10), and DCM-CLSTN1 OE (*n* = 13). Three rats were excluded because of fatal bleeding induced by carotid artery cannulation during hemodynamic measurements (DCM-WT group, *n* = 1; Con-CLSTN1 OE group, *n* = 1; DCM-CLSTN1 OE, *n* = 1). Supplement Table 1 lists the body weight and organ weights of each case. The weight of the left ventricle decreased marginally after CLSTN1 overexpression, without a statistical difference.

**Fig. 6:**
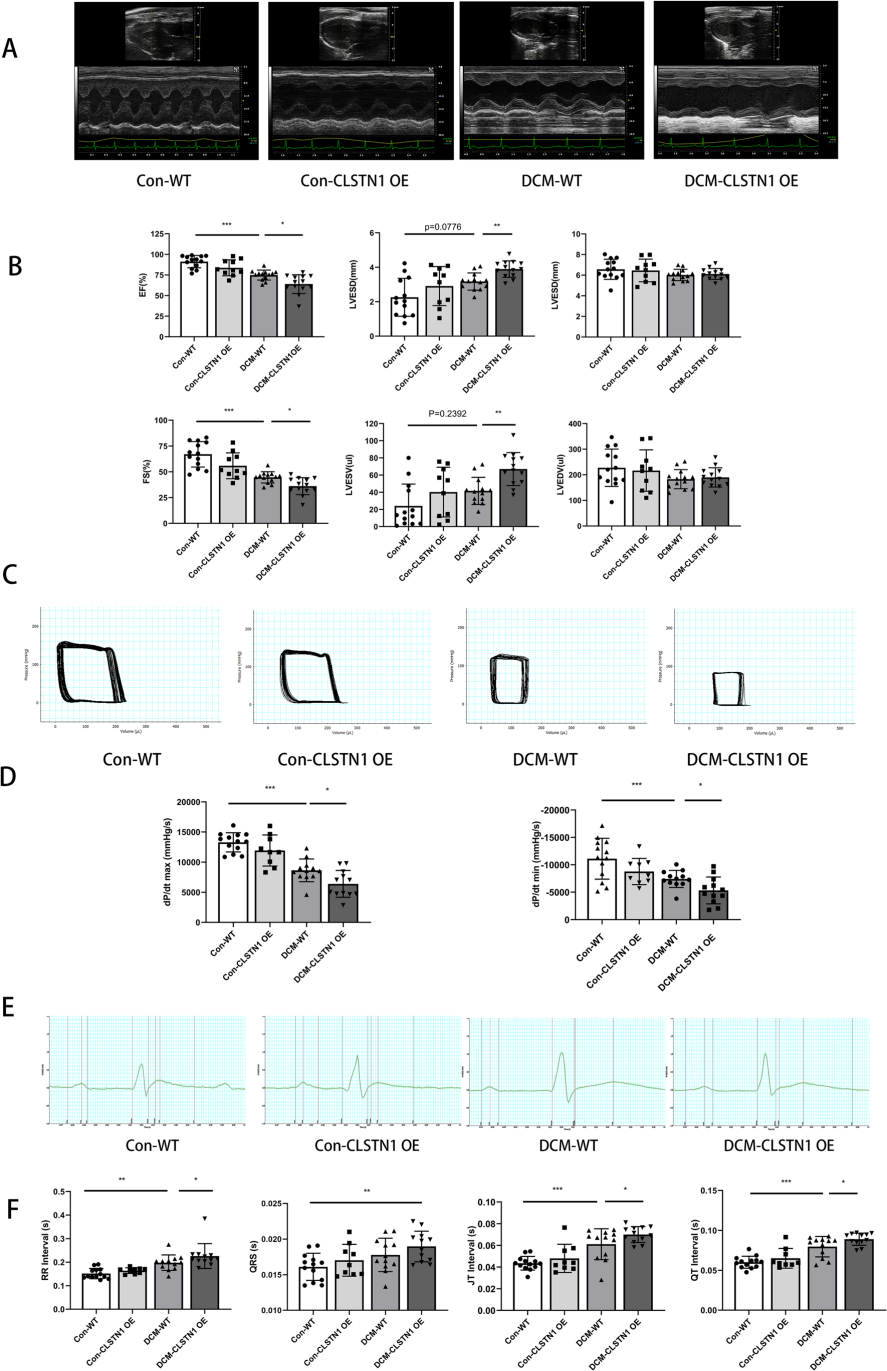
CLSTN1 overexpression aggravates impaired heart function. In the DCM model induced by doxorubicin injection, CLSTN1 overexpression aggravates DCM as assessed by electrocardiogram, hemodynamics, and electrocardiogram. **(A)** The typical ultrasound images obtained from the experiments. **(B)** Statistical analysis of EF, FS, LVESD, LVESV. **(C)** The typical pressure-volume loop (PV loop) of the left ventricle. The P-V loop shifted right and decreased in size after CLSTN1 overexpression. **(D)** Statistical analysis of LV dp/dt max and dp/dt min. (E) The typical electrocardiograms (ECG). **(F)** Statistical analysis of RR interval, heart rates, JT interval, and QT interval in electrocardiograms. Con, control, saline treatment; DCM, doxorubicin-induced dilated cardiomyopathy; WT, wide type; CLSTN1 OE, CLSTN1 overexpression. ***P < 0.001, **P < 0.01, *P < 0.05

The results of the echocardiographic analysis indicated a drastic reduction in fractional shortening and ejection fraction alterations and a significant increase in left ventricular end-systolic diameter and end-systolic volume following CLSTN1 overexpression in the myocardium, while no significant changes in left ventricular end-diastolic diameter and end-diastolic volume could be observed (Fig. 6A and B; Supplement Table 2). Cardiac function was also estimated as a hemodynamic parameter. In the CLSTN1 overexpression groups, a rightward shift and shrinking of the pressure-volume (P-V) loop was observed along with a decreased maximum +dP/dt (+dP/dt max) and −dP/dt (−dP/dt max) and increased tau value in the CLSTN1 overexpression groups, all of which indicated impaired cardiac muscle contraction capacity (Fig. 6C and D; Supplement Table 3). Additionally, CLSTN1 overexpression exacerbated the DCM-induced arrhythmia and lead to prolonged RR, QRS, JT, and QT intervals, according to ECG detection (Fig. 6E and F; Supplement Table 4). These results demonstrated that CLSTN1 overexpression in cardiomyocytes impaired cardiac systolic contractile ability and damaged pump function, aggravating DCM.

### Knockdown of CLSTN1 Rescues Cardiomyocyte Toxicity In Vitro

**Fig. 7:**
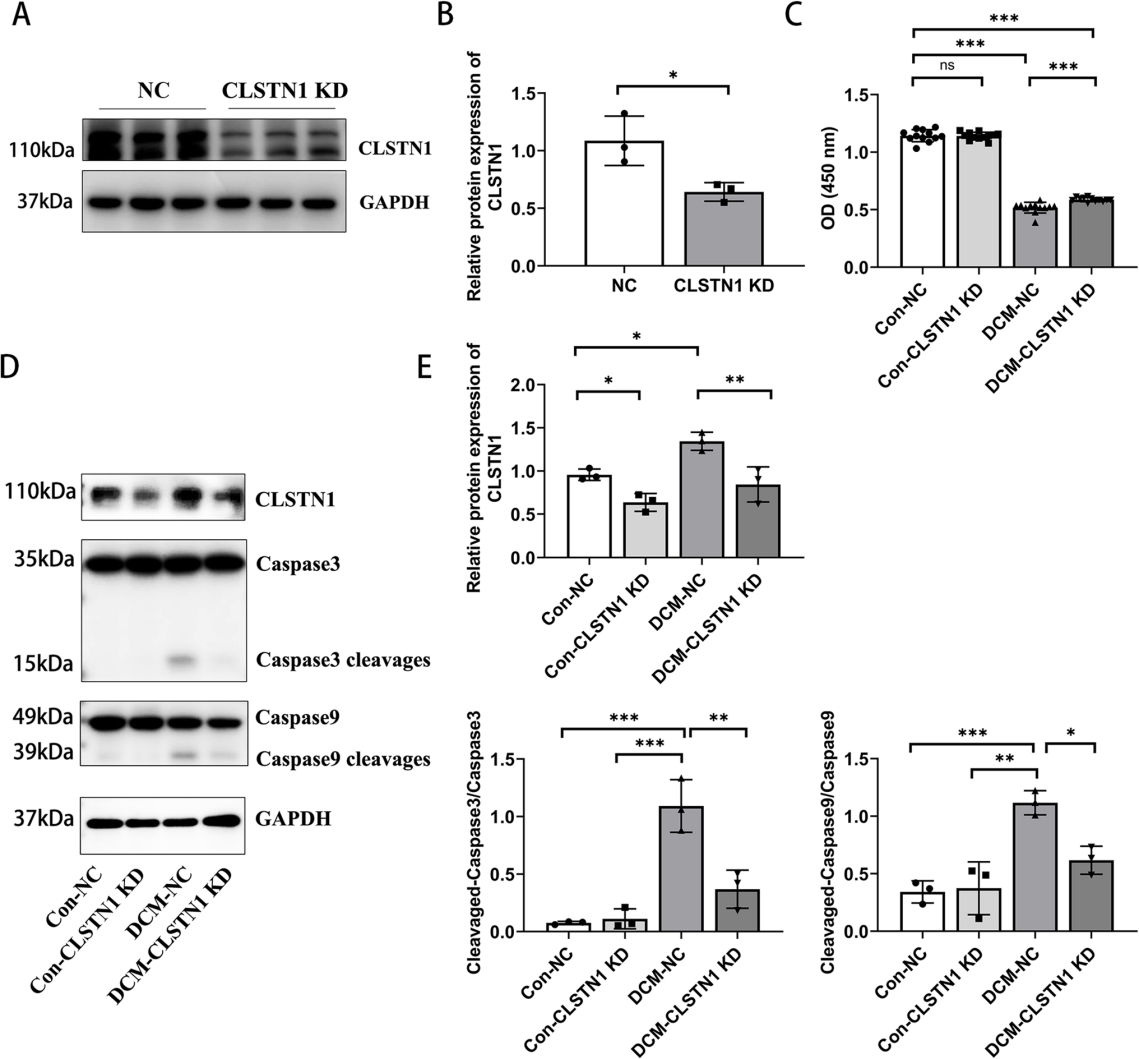
Knockdown of CLSTN1 rescues cardiomyocyte toxicity in vitro.** (A)** H9c2 cells were transfected with CLSTN1-KD lentiviruses or an empty control for 48 h. Protein lysates were used for immunoblots to assess CLSTN1 protein level. **(B)**Statistical results for relative protein expression of CLSTN1 in NC and CLSTN1 KD cells. **(C)** H9c2 cells with or without CLSTN1 knockdown were detected by CCK8 assay for cellular viability. **(D)** Western blot of effects in CLSTN1 knockdown on apoptosis protein expression. **(E)** Statistical results for relative protein expression of CLSTN1, cleaved caspase 3, and cleaved caspase 9. ***P < 0.001, **P < 0.01, *P < 0.05 versus control

In vitro, we decreased CLSTN1 expression by lentivirus-mediated shRNA in cultured H9c2 cells and the transfection efficiency was examined by western blot. Compared with cells transfected with empty lentivirus vectors (NC cells), the protein level of CLSTN1 in CLSTN1-knock-down cells (KD cells) was greatly decreased (Fig. 7A and B). Both NC cells and KD cells were treated with 1 μM Dox for 24 h, after which the CCK8 contents were evaluated. Our data revealed that the Dox-treated NC cells had a much larger percentage of damaged cells, whereas CLSTN1 knockdown had a significantly lower percentage of damaged cells. In addition, the damage rate of the KD cells did not differ from that of NC cells (Fig. 7C). Western blot analysis was performed to investigate the activation of caspase-3/caspase-9. As presented in Fig. 7D and E, cleaved caspase-3/caspase-9 was found to be significantly upregulated in cells treated with Dox, and this effect was attenuated by the knockdown of CLSTN1. At the same time, this knockdown without Dox had no significant impact on the level of cleaved caspase-3/caspase-9.

### CLSTN1 Influences PI3K-Akt Pathway and Calcium Pathway

**Fig. 8:**
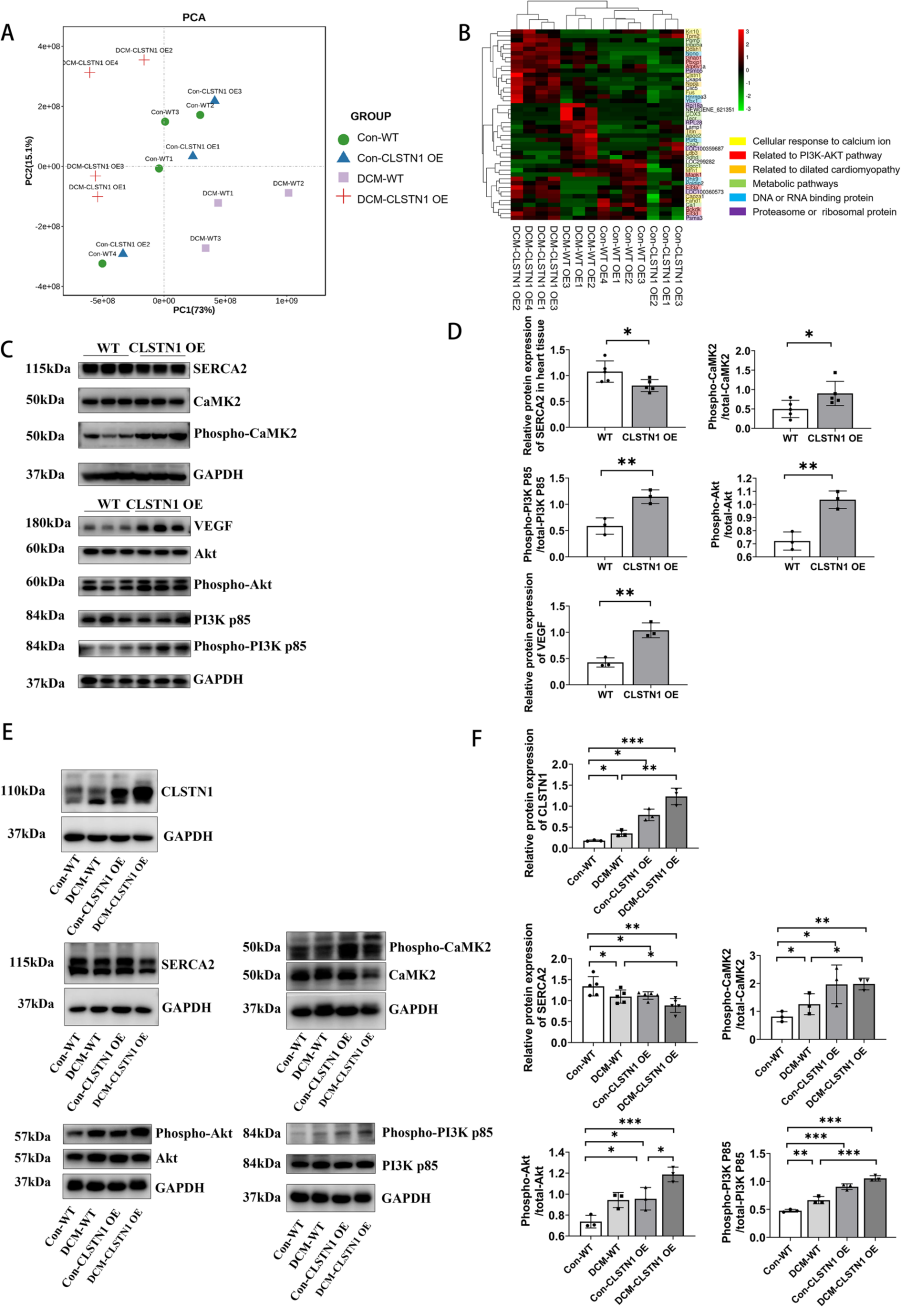
Putative molecular mechanisms.** (A)** Principal component analysis (PCA) of all four groups. On the first two principal components, DCM-CLSTN1 clusters far away from DCM-WT. **(B)** Heat map for the enriched proteins. The green color represents downregulation, while the red represents upregulation. Genes are linked with potential biological function by KEGG, the primary gene function and pathways are labeled using the corresponding color in this figure. **(C)** Western blot results of changes in CLSTN1 overexpression on PI3K-Akt and calcium pathway. **(D)** Western blot statistical results for relative expression of proteins in the PI3K-Akt and calcium pathway. **(E)** Western blot of effects of PI3K-Akt and calcium pathway in the CLSTN1 overexpression in the DCM model. **(F)**Statistical results for relative protein expression in the DCM model on PI3K-Akt and calcium pathway. **P < 0.01, *P < 0.05 versus wild type group

To further explore the molecular interaction of CLSTN1, proteomic analysis was performed for heart samples from the four groups previously described. CLSTN1 overexpression samples were found to be quite different from the wild-type ones (Fig. 8A). A total of 3000 proteins were identified, and 44 of these proteins had both *P* values < 0.05, and fold change > 2 or < 0.5. A heat map based on these 44 differentially expressed genes was generated. According to KEGG, the protein expression patterns changed significantly among the different groups, and the proteins with significantly altered expression were primarily involved in the cellular response to calcium ions, PI3K-Akt pathway, DCM, metabolic pathways, DNA or RNA binding, and components of proteasome or ribosome (Fig. 8B).

Based on the results of the western blot assay, we found that overexpression of CLSTN1 increased the protein levels of VEGF and enhanced the phosphorylation levels of Akt and PI3K p85. These findings suggest that CLSTN1 overexpression can enhance protein phosphorylation in the PI3K-Akt pathway and may, thereby, affect heart structure and function. We also found that the CLSTN1 overexpression group showed higher levels of phosphorylation of CaMK2 and lower levels of SERCA2. (Fig. 7C and D). Furthermore, the DCM group showed elevated levels of Akt and PI3K p85 phosphorylation, while CLSTN1 overexpression raised these protein levels even further. The DCM group also showed increased phosphorylation of CaMK2 and downregulated SERCA2, which was further altered when CLSTN1 was overexpressed (Fig. 8E and F).

## Discussion

Dox is a frequently used chemotherapeutic agent in oncology. However, its use in clinics is constrained by the severe cardiac side effects, such as DCM, that it produces [1]. Therefore, understanding the molecular mechanism of Dox-induced DCM could be of significant importance for the clinical diagnosis and treatment of the disease. In this study, we investigated the role of CLSTN1 and the underlying mechanism in Dox-induced cardiotoxicity. Our findings showed that CLSTN1 protein expression was significantly increased in Dox-treated rat hearts and H9c2 cardiomyoblasts. Overexpression of CLSTN1 caused impaired cardiac systolic contractile ability and damaged pump function, which promotes DCM, while the knockdown of CLSTN1 inhibited Dox-induced apoptosis and reduced cardiotoxicity in cardiomyoblasts. These observations revealed that CLSTN1 might play an important role in the progression of Dox-induced DCM and could be a potential therapeutic target to prevent the development of Dox-induced DCM.

Calcium plays an important role in cell signaling, nerve impulse transmission, muscle contraction, and maintaining calcium homeostasis in mammals. In muscle contraction, these ions help catalyze troponin and tropomyosin, located in the thin filament [22]. CLSTN1 is a type I transmembrane protein and a member of the cadherin superfamily. It possesses the ability to bind to Ca^2+^ with its cytoplasmic domain, regulate calcium-mediated signaling, and is thus essential for maintaining calcium homeostasis [4, 5]. It has been reported that CLSTN1 could regulate the CaMK2 pathway via the L-type Ca^2+^ channel to promote mitochondrial defects and axon degeneration [23]. Calsyntenin-3 (CLSTN3), which belongs to the calsyntenins superfamily and is similar to CLSTN1 in their molecular structures and calcium-binding capacity, has also been reported to modulate calcium-mediated postsynaptic signaling in neuronal growth [4, 24, 25]. In addition, cadherin dysfunction, a major pathology linked to neuromuscular disorders and cardiomyopathy, typically affects muscular tissues [26]. Although predominantly expressed in the postsynaptic membranes of pyramidal neurons, CLSTN1 can be detected in many other tissues, including the heart, skeletal muscle, kidneys, and placenta [5, 6]. However, the role of CLSTN1 in the cardiovascular system is poorly understood as it is a widely expressed gene in numerous tissues and organs. Based on proteomic analyses and RNA expression profiling, CLSTN1 has previously been identified as a potentially pathogenic gene in ischemic cardiomyopathy and coronary artery disease in human samples [12, 13]. However, these results were not studied in-depth.

In this study, we discovered that CLSTN1 protein expression was drastically increased in Dox-induced DCM rats and H9c2 cells. Using cardiac-specific CLSTN1 overexpression rats, we confirmed that this protein could aggravate deterioration in cardiac contractility and deficits in systolic function and interfere with the electrical activity of the heart, thus aggravating doxorubicin-induced cardiotoxicity. In vitro, with the help of CLSTN1-KD cells, we first demonstrated that targeting CLSTN1 can rescue Dox-induced cardiomyocyte toxicity. Dox treatment induces decreased cell viability and apoptosis in cardiomyocytes [27]. A previous study indicates that Dox-induced apoptosis is mediated by the intrinsic signaling cascade, ultimately activating caspase-3 and leading to cell death [28]. In line with these former studies, we found that Dox significantly activated caspase-3/caspase-9 and decreased viability in H9c2 cells. Moreover, CLSTN1 downregulation reversed the activation of caspase-3 and caspase-9, and attenuated the cell injury induced by Dox. To the best of our knowledge, this is the first time the pathological function of CLSTN1 in the cardiovascular system has been reported.

To further examine the pathogenic mechanism underpinning CLSTN1, we performed proteomic analysis to investigate the signaling pathways associated with it. In this study, we detected 44 differentially expressed proteins that were involved in the cellular response to calcium ions, PI3K-Akt pathway, DCM, metabolic pathways, DNA or RNA binding, and as components of the proteasome or ribosome. Researchers have shown that CLSTN1 regulates the PI3K-Akt pathway, a pathway that has been proven to be an important regulator in Dox-induced cardiac injury [29]. Therefore, we focused on how CLSTN1 overexpression affected protein expression in the PI3K-Akt pathways. Our findings verified previous research that the overexpression of CLSTN1 enhanced the phosphorylation levels of PI3K/Akt and that Dox increased the expression in PI3K/Akt phosphorylation. Furthermore, our findings reported here show that the overexpression of CLSTN1 can further enhance the phosphorylation levels of Akt and PI3K p85 induced by Dox.

A previous study also reported that CLSTN1 can bind to Ca^2+^ with its cytoplasmic domain and may modulate the Ca^2+^-mediated postsynaptic signaling pathway [30, 31]. SERCA2, a sarcoplasmic reticulum Ca^2+^-ATPase pump, is a calcium pump protein that can mediate sarcoplasmic reticulum Ca^2+^ cycling and Ca^2+^ re-uptake. Some have reported that decreased SERCA2 activity has been detected in DCM and heart failure [32]. A previous study showed that CaMK2, a Ca^2+^/calmodulin-dependent protein kinase II, is also involved in DCM. The expression and phosphorylation of CaMK2 are higher in failing myocardium than that in a healthy myocardium [33]. Our findings verified here that Dox enhanced the phosphorylation levels of CaMK2 as well as the downregulation of SERCA2, which is consistent with the findings of earlier research. As previously described, CLSTN1 can bind to calcium ions and regulate calcium-mediated signaling [4, 5, 23]. In our study here, overexpressing CLSTN1 further elevated CaMK2 phosphorylation, and downregulated SERCA2 in myocardial cells, as confirmed by western blotting, suggesting that CLSTN1 may be involved in the regulation of calcium signaling and the development of Dox-induced DCM. Although detailed mechanistic research is required, we confirm that our study provides a significant clue toward the detrimental role that CLSTN1 plays in Dox-induced DCM.

## Limitations

Some limitations in our study should be noted. First, a CLSTN1 knockdown rat model is required to further demonstrate the rescue effect of targeting CLSTN1 on cardiac function. Second, the changes in CLSTN1 expression in patients with Dox-induced DCM needs further investigation. Third, some other potential pathways screened by our proteomic analyses should be further investigated, such as the metabolism pathway.

## Conclusion

These results demonstrate that CLSTN1 plays an important role in the pathogenesis of Dox induced DCM and may affect heart function via regulating expression of SERCA2 and phosphorylation of CaMK2 and PI3K-Akt. This finding may provide new insights into the underlying molecular mechanisms of Dox-induced DCM and may provide a new therapeutic target for diagnosis and treatment.

### Supplementary Information

ESM 1 (DOCX 37 kb)

## Data Availability

The original contributions presented in the study are included in the article/Supplementary Material. Raw data from mass spectrum were deposited in iProx (http://www.iprox.org) with accession number IPX0004414001. Further inquiries can be directed to the corresponding author.

## Abbreviations

CLSTN1: calsyntenin-1
DCM: dilated cardiomyopathy
Dox: doxorubicin
LV: Left ventricular
LVAWTd: the diastolic left ventricular anterior wall thickness
LVAWTs: systolic left ventricular anterior wall thickness
LVPWTd: diastolic left ventricular posterior wall thickness
LVPWTs: systolic left ventricular posterior wall thickness
LVEDD: left ventricular end-diastolic diameter
LVESD: left ventricular end-systolic dimension
LVEDV: left ventricular end-diastolic volume
LVESV: left ventricular end-systolic volume
EF: LV ejection fraction
FS: fractional shortening
qPCR: quantitative real-time RT-PCR
DAPI: 4',6-diamidino-2-phenylindole
GAPDH: glyceraldehyde-3-phosphate-dehydrogenase
PCA: principal component analysis
ECG: electrocardiogram
HE: Hematoxylin and eosin
