# Supplementary material for: Calsyntenin-1 Promotes Doxorubicin-induced Dilated Cardiomyopathy in Rats
Source: Cardiovasc Drugs Ther. 2022 Nov 9;38(2):237–52. doi: 10.1007/s10557-022-07389-x (PMC10959838; doi:10.1007/s10557-022-07389-x)
Supplement: Supplementary file 1 — (DOCX 37 kb) [file 10557_2022_7389_MOESM1_ESM.docx]

**Supplement Table 1.**

|  | **Con** | | | | **DCM** | | | |  |
| --- | --- | --- | --- | --- | --- | --- | --- | --- | --- |
|  | **WT** | | **CLSTN1 OE** | | **WT** | | **CLSTN1 OE** | |  |
| body weight (g) | | 391.5±48.12 | | 377.8±40.77 | | 224.9±27.42* | | 232.0±23.13* | |
| heart weight (g） | | 1.055±0.1426 | | 0.9296±0.1048 | | 0.4042±0.2315* | | 0.3268±0.0444* | |
| heart weight /body weight（‰） | | 2.725±0.1365 | | 2.463±0.1045* | | 1.565±0.3803* | | 1.426±0.1496* | |
| LV weight (g) | | 0.5593±0.08115 | | 0.4886±0.07703 | | 0.1735±0.1238* | | 0.1280±0.02472* | |
| LV/body weight（‰） | | 1.444±0.08226 | | 1.292±0.1283 | | 0.6578±0.2202* | | 0.5627±0.1261* | |
| IS (g) | | 0.2544±0.03935 | | 0.2206±0.03184 | | 0.5817±0.11002* | | 0.6074±0.06201* | |
| IS weight /body weight（‰） | | 0.6594±0.07695 | | 0.5847±0.05761 | | 2.488±0.6731* | | 2.652±0.1484* | |
| RV weight (g) | | 0.2410±0.0436 | | 0.2205±0.02089 | | 0.1537±0.04634* | | 0.1526±0.02777* | |
| RV weight /body weight（‰） | | 0.6220±0.06972 | | 0.5858±0.04355 | | 0.6182±0.09217 | | 0.6627±0.08619 | |
| lung weight (g) | | 1.360±0.09165 | | 1.297±0.1064 | | 1.181±0.1373 | | 1.269±0.2203 | |
| lung weight /body weight（‰） | | 3.876±0.2457 | | 3.700±0.4553 | | 5.032±0.8837* | | 5.610±1.340* | |
| Liver(g) | | 13.18±1.306 | | 13.85±1.575 | | 11.54±2.015* | | 10.50±1.567* | |
| Liver weight /body weight（‰） | | 34.20±2.232 | | 36.94±4.2050 | | 48.52±6.734* | | 45.84±6.008* | |
| kidney(g) | | 1.242±0.1527 | | 1.333±0.1965 | | 0.9906±0.1617* | | 1.015±0.1204* | |
| Left kidney/body weight（‰） | | 3.218±0.2610 | | 3.150±0.6625 | | 4.174±0.5885* | | 4.447±0.5299* | |
| spleen weight (g) | | 0.6535±0.09173 | | 0.7078±0.1146 | | 0.4097±0.1830* | | 0.4048±0.1549* | |
| spleen weight /body weight（‰） | | 1.722±0.3745 | | 1.927±0.4507 | | 1.6779±0.5518 | | 1.759±0.6256 | |

**Body weight and organ weights.** Body weight and organ weights were summarized. Organ weights were normalized to body weight. LV, left ventricle; IS, interventricular septum; RV, right ventricle. *p<0.05 versus Con-WT group.

**Supplement Table 2.**

|  | **Con** | | **DCM** | |
| --- | --- | --- | --- | --- |
|  | **WT** | **CLSTN1 OE** | **WT** | **CLSTN1 OE** |
| EF(%) | 91.22±7.43 | 83.71±9.65* | 74.89±6.04* | 63.96±11.36*# |
| FS(%) | 66.98±12.29 | 55.86±12.51 | 44.63±5.46* | 36.11±8.24*# |
| LVEDV | 227.48±72.85 | 216.43±81.10 | 182.87±37.28 | 189.84±37.72 |
| LVESV | 24.04±25.48 | 40.19±28.92 | 41.56±15.77 | 67.05±19.19*# |
| LVAWTd(mm) | 2.15±0.36 | 2.08±0.34 | 1.77±0.19* | 1.65±0.12* |
| LVAWTs(mm) | 4.04±0.48 | 3.54±0.54 | 2.89±0.31 | 2.63±0.27 |
| LVEDD(mm) | 6.57±0.97 | 6.47±1.10 | 5.91±0.55 | 6.12±0.53 |
| LVESD(mm) | 2.26±1.10 | 2.91±1.13 | 3.17±0.50 | 3.89±0.48 |
| LVPWTd(mm) | 2.77±0.57 | 2.35±0.48* | 2.10±0.37* | 2.09±0.44* |
| LVPWTs(mm) | 4.27±0.69 | 3.55±0.39* | 3.02±0.49* | 2.78±0.31* |

**Echocardiographic analysis of cardiac function.** Echocardiography was used to detect the rat LV cardiac functions. EF, Ejection Fraction; FS, LV fractional shortening; LVEDV, left ventricular end-diastolic volume; LVESV, left ventricular end-systolic volume; LVAWTd, diastolic left ventricular anterior wall thickness; LVAWTs, systolic left ventricular anterior wall thickness; LVEDD, left ventricular end-diastolic diameter; LVESD, left ventricular end-systolic diameter; LVPWTd, end-diastolic left ventricular posterior wall thickness; LVPWTs, end-systolic left ventricular posterior wall thickness. *p<0.05 versus Con-WT group. #p<0.05 versus DCM-WT group.

**Supplement Table 3.**

|  | **Con** | | **DCM** | |
| --- | --- | --- | --- | --- |
|  | **WT** | **CLSTN1 OE** | **WT** | **CLSTN1 OE** |
| SW (mmHg*µL) | 19289.23±5515.21 | 15689.33±5417.7 | 9187.75±2684.5* | 9007.42±4759.14* |
| CO (µL/min) | 77399.23±21036.56 | 62176.67±26303.98 | 35007.50±7408.17* | 35156.67±12914.43* |
| SV (µL) | 189.25±44.55 | 166.27±62.03 | 115.65±24.00* | 125.64±38.7* |
| Vmax (µL) | 255.73±69.86 | 224.72±89.26 | 161.57±32.28* | 200.18±43.57* |
| Vmin (µL) | 46.04±10.53 | 58.45±42.65 | 68.09±37.71 | 74.55±20.83 |
| Ves (µL) | 69.61±39.56 | 62.83±44.45 | 48.91±12.55 | 79.95±20.55 |
| Ved (µL) | 246.15±68.71 | 212.94±88.22 | 151.20±32.77* | 187.39±38.48 |
| Pmean (mmHg) | 58.8±12.11 | 59.72±14.53 | 49.23±8.72 | 44.67±13.23 |
| Pdev (mmHg) | 127.47±15.96 | 119.5±15.36 | 101.86±13.86* | 90.27±19.96* |
| Pes (mmHg) | 115.56±24.5 | 111.80±24.21 | 97.38±12.84* | 86.09±20.9* |
| Ped (mmHg) | 3.26±3.08 | 4.55±4.18 | 1.72±1.53 | 2.15±2.48 |
| HR (bpm) | 407.35±48.2 | 368.91±27.45 | 305.74±38.46* | 279.13±45.21* |
| Ea (mmHg/µL) | 0.65±0.22 | 0.78±0.24 | 0.90±0.26* | 0.74±0.20 |
| PowMax (mmHg*µL/s) | 87296.15±56340.42 | 160371.11±161358.26 | 25899.75±19223.79 | 92488.33±110577.18 |
| dP/dt max (mmHg/s) | 13284.62±1595.00 | 11928.78±2575.15 | 8636.42±1885.66* | 6385.75±2224.88*# |
| dP/dt min (mmHg/s) | -11120.92±3720.56 | -8787.56±2388.87 | -7516.17±1499.59* | -5307.33±2411.43*# |
| dV/dt max (µL/s) | 4052.15±866.07 | 4818.78±1992.91 | 2639.17±595.19* | 3592.08±1843.73 |
| dV/dt min (µL/s) | -4662.08±1411.10 | -5442.67±4773.49 | -1999±526.43* | -3076.42±1507.11 |
| P@dV/dt max (mmHg) | 4.14±4.91 | 14.27±23.65* | 1.29±3.80 | 4.11±7.17 |
| P@dP/dt max (mmHg) | 86.87±18.32 | 81.55±17.94 | 70.30±10.43* | 61.41±16.02* |
| V@dP/dt max (µL) | 233.19±58.34 | 200.57±63.73 | 143.98±32.85* | 182.08±46.76* |
| Tau | 8.91±2.05 | 10.64±1.24 | 12.15±2.58* | 14.22±2.84*# |

**Summary of hemodynamic data.** SW, Stroke Work; CO, Cardiac Output; SV, Stroke Volume; Vmax , Maximum Volume; Vmin, Minimum Volume; Ves, End-systolic Volume; Ved, End-diastolic Volume; Pmean, Mean Pressure; Pdev, Developed Pressure; Pes, End-systolic Pressure; Ped, End-diastolic Pressure; HR, Heart Rate; Ea, Arterial elastance; PowMax, Maximum Power; dP/dt max, Maximum value of the pressure derivative (dP/dt) during a loop; dP/dt min, Minimum value of the pressure derivative (dP/dt) during a loop; dV/dt max, Maximum value of the volume derivative (dV/dt) during a loop; dV/dt min, Minimum value of the volume derivative (dV/dt) during a loop; P@dV/dt max, Value of pressure at the point where the maximum volume derivative (dV/dt max) occurs; P@dP/dt max (mmHg), Value of pressure at the point where the maximum pressure derivative (dP/dt max) occurs; V@dP/dt max (µL), Value of volume at the point where the maximum pressure derivative (dP/dt max) occurs; V@dP/dt min, Value of volume at the point where the minimum pressure derivative (dP/dt min) occurs; Tau, the time-constant of isovolumic relaxation, using Weiss Model. *p<0.05 versus Con-WT group. #p<0.05 versus DCM-WT group.

**Supplement Table 4**

|  | **Con** | | **DCM** | |
| --- | --- | --- | --- | --- |
|  | **WT** | **CLSTN1 OE** | **WT** | **CLSTN1 OE** |
| RR Interval (s) | 0.152±0.0220 | 0.164±0.0121 | 0.198±0.0330* | 0.226±0.0528*# |
| Heart Rate (BPM) | 403.43±54.72 | 368.08±27.77 | 311.15±52.11* | 274.78±44.45* |
| PR Interval (s) | 0.0485±0.00699 | 0.0504±0.00516 | 0.0516±0.00769 | 0.054±0.01106 |
| P Duration (s) | 0.0174±0.00698 | 0.0183±0.00300 | 0.0165±0.00314 | 0.0176±0.00422 |
| QRS Interval (s) | 0.0161±0.0019 | 0.0170±0.0022 | 0.0178±0.0023 | 0.0190±0.0022* |
| QT Interval (s) | 0.0601±0.0750 | 0.0650±0.0124 | 0.0796±0.0128* | 0.0889±0.00762*# |
| QTc (s) | 0.155±0.0160 | 0.161±0.0295 | 0.181±0.0313* | 0.189±0.0161* |
| JT Interval (s) | 0.0435±0.063 | 0.0480±0.0130 | 0.0611±0.0142* | 0.0700±0.00739*# |
| T peak Tend Interval (s) | 0.0318±0.0631 | 0.0380±0.0105 | 0.0382±0.0112 | 0.0420±0.0678* |
| P Amplitude (mV) | 0.112±0.0624 | 0.130±0.0287 | 0.118±0.0567* | 0.0849±0.0564* |
| Q Amplitude (mV) | -0.489±0.113 | -0.0188±0.0294 | -0.0162±0.0236 | -0.0184±0.0162 |
| R Amplitude (mV) | 0.636±0.222 | 0.692±0.215 | 1.07±0.230* | 1.05±0.306* |
| S Amplitude (mV) | -0.228±0.131 | -0.315±0.119 | -0.309±0.184* | -0.119±0.111*# |
| ST Height (mV) | 0.126±0.162 | 0.145±0.0812 | 0.0547±0.0458* | 0.0393±0.0296* |
| T Amplitude (mV) | 0.202±0.109 | 0.247±0.0896 | 0.259±0.0922 | 0.216±0.0625 |

**Electrocardiogram.** The results of rat limb lead electrocardiogram. *p<0.05 versus Con-WT group. #p<0.05 versus DCM-WT group.
